# Protective effect of Sam-Hwang-Sa-Sim-Tang against hepatic steatosis in mice fed a high-cholesterol diet

**DOI:** 10.1186/1472-6882-13-366

**Published:** 2013-12-23

**Authors:** Tae-Gue Ahn, Joo-Young Lee, Se-Yun Cheon, Hyo-Jin An, Yoon-Bum Kook

**Affiliations:** 1 Department of Pharmacology, College of Oriental Medicine, Sangji University, Wonju-si, Gangwon-do 220-702, Republic of Korea; 2 Department of Presctiption, College of Oriental Medicine, Sangji University, Wonju-si, Gangwon-do 220-702, Republic of Korea

**Keywords:** Sam-Hwang-Sa-Sim-Tang (SHSST), Liver steatsosis, High-cholesterol diet (HCD)

## Abstract

**Background:**

Sam-Hwang-Sa-Sim-Tang (SHSST) is a traditional Oriental medication that has been commonly used in Korea for the treatment of hypertension, insomnia, and chest pain. In addition, some studies reported that administration of SHSST results suppression of hyperlipidemia in rats or lowering lipid plasma level such as total cholesterol (TC). Those results made us find and demonstrate positive effect of SHSST much more. The aim of the current study was to examine whether SHSST exerts an effect against hepatic steatosis and two type of SHSST has different efficacy on liver steatosis.

**Methods:**

Total 40 mice were divided randomly and equally into 4 groups: a normal diet (CON) group, high-cholesterol diet (HC) group, and treatment groups fed a high-cholesterol diet (HCD) with a 30% or 80% ethanol extract of SHSST (SHSST-L and SHSST-H, respectively). The HCD was given for 9 weeks. The SHSST-treated groups were orally administered SHSST at a dose of 150 mg/kg, whereas the other groups received physiological saline.

**Results:**

SHSST administration to mice resulted in a decline in serum levels of total cholesterol and low-density lipoprotein. Histological examination showed that lipid droplets were smaller in the SHSST-treated group than in the HC group. At the protein level, expression of sterol regulatory element-binding protein 2 (SREBP-2) was suppressed by SHSST. In addition, the mRNA expression of cholesterol metabolism-related molecules such as SREBP-2, liver X receptor (LXR), low-density lipoprotein receptor (LDLR), and 3-hydroxy-3methylglutary-CoA (HMG-CoA) was also suppressed in SHSST-treated groups in the liver. In the aorta tissue, SHSST decreased the expression of tumor necrosis factor-α (TNF-α), interleukin-6 (IL-6), intercellular adhesion molecule-1 (ICAM-1), vascular cell adhesion molecule-1(VCAM-1), transforming growth factor (TGF)-β1, and fibronectin.

**Conclusions:**

The present study indicates that SHSST protects against liver steatosis and protects vessels against inflammation arising from excessive ingestion of cholesterol. These findings may also suggest that SHSST could be used as an adjuvant remedy for protection against liver steatosis.

## Background

Metabolic syndrome is a group of conditions that increase the risk of diabetes, cardiovascular diseases, and carcinogenesis [1]. Genetic and environmental factors are considered to promote the development of metabolic syndrome. In addition, obesity, diabetes, and non-alcoholic fatty liver disease (NAFLD) are common in metabolic syndrome [2,3]. NAFLD frequently causes abnormal liver function, and one of the characteristic features of NAFLD is hepatic steatosis [4]. The role of dietary cholesterol, with the subsequent increased hepatic esterification of cholesterol and its association to hepatic triglyceride accumulation, is a new paradigm for hepatic steatosis [5]. Although hepatic steatosis can proceed to end-stage liver disease, ideal treatment for this disease has not yet been established. Therefore, prevention of hepatic steatosis may be useful for populations at risk of advanced liver disease [6,7].

Sam-Hwang-Sa-Sim-Tang (SHSST) is composed of 3 herbs; *Coptidis rhizoma* of the Ranunculaceae family (rhizomes of *Coptis chinensis*; CC), *Scutellariae radix* of the Labiatae family (roots of *Scutellaria baicalensis*; SB), and *Rhei rhizoma* of the Polygonaceae family (rhizomes of *Rheum officinale*; RO). In traditional Korean medicine, SHSST has been commonly prescribed for hypertension, insomnia, and chest pain [8-10]. Recent studies have also reported various beneficial effects of SHSST, including anti-inflammatory, antihypertensive, antiatherogenic, and antioxidant effects [11-14]. In addition, there were studies that reported SHSST-administrated rats showed the tendency of decline of several lipid plasma levels including total cholesterol and administration of SHSST suppressed the development of hyperlipememia [10,11]. Generally, liver is considered as central organ in lipid metabolism [15,16]. Therefore, those previous studies made us think SHSST might show those positive effects because of some positive connection with metabolic role of liver. Plus, several studies have suggested that the efficacy of herbs could differ depending on the type of solvent, e.g., water, methanol, or ethanol, or the concentration of solvent used to extract the active ingredient [17-20].

Taking into all those facts account, we decided to study the positive effect of SHSST related with lipid metabolism specifically in liver. We finally hypothesized that SHSST might have a protective effect against hepatic steatosis induced by a high-cholesterol diet (HCD) because hepatic steatosis induced by HCD is basic features of NAFLD and it is also one of general pathological status closely related with lipid metabolism. In addition, we also hypothesized the efficacy of SHSST against hepatic steatosis may differ depending on the concentration of ethanol used to extract active ingredients from SHSST. In addition, because the protective effect of SHSST against hepatic steatosis is not known, we decided to study the effects of SHSST in mice with hepatic steatosis induced by HCD. To our knowledge, this is the first study to evaluate the efficacy of SHSST against liver steatosis according to the concentration of ethanol used for extraction of SHSST.

## Methods

### Reagents

CF, SG, and RB were purchased from Omniherb Co. Ltd (Daegu, Republic of Korea). The normal diet and HCD were obtained from Research Diets (New Brunswick, NJ, USA). The other reagents were purchased from Sigma-Aldrich (St. Louis, MO, USA), unless otherwise specified in the text.

### Preparation of SHSST

SHSST consists of CC, SB, and RO. Each herb was used in a ratio of 1:1:1 (300 g: 300 g:300 g). The herbs had a moisture content of <11% by weight and were air-dried. The combination of herbs was subjected to extraction with 30% or 80% (v/v) ethanol in water at 60°C for 8 h. The extracts were filtered with 10 μM cartridge paper, and the ethanol was removed via vacuum rotary evaporation (Eyela, Japan). The concentrates were freeze-dried, and the yields were 13% and 15% for the 30% and 80% ethanol extracts, respectively. The powders were dissolved in distilled water for the experiments, and the residual powders were stored at −20°C.

### Animal experiments

Forty male C57BL/6 J mice weighing 18–19 g at the age of 3 weeks were purchased from Daehan Biolink (Daejeon, Republic of Korea). The animals were all maintained in accordance with conditions recommended according to the National Institutes of Health guidelines. The Institutional Animal Care Committee of Sangji University is according to the NIH guidelines. This study was submitted recommended by the Institutional Animal Care Committee of Sangji University (reg. no. 2012–3). The mice were adapted to the feeding conditions for 2 weeks. They were then given free access to food and tap water for 9 weeks and were housed under a 12 h light/dark cycle at a constant temperature of 22°C ± 2°C and relative humidity of 55% ± 10%. The mice were randomly separated into 6 groups of 10 mice each: the normal diet (CON) group, HCD (HC) group, and treatment groups fed HCD with the 30% or 80% ethanol extract of SHSST (L and H, respectively). Mice except CON group consumed HCD (D12108; Research Diets Inc., New Brunswick, New Jersey, USA) containing 20.1% saturated fat, 1.37% cholesterol, and 0% sodium cholate. The SHSST-treated groups were orally administered with SHSST at a dose of 150 mg/kg. The other groups were administered the same volume of physiological saline. Body weight and dietary intake were recorded every week. At the end of this period, the animals were fasted overnight. The following day, they were anesthetized with Zoletil (Virbac, Carros Cedex, France), and blood samples were then collected by cardiac puncture. The liver and aorta tissue were then excised, rinsed, weighed, and directly stored at −80°C until analysis.

### Serum analysis

Serum concentrations of total cholesterol (TC), low-density lipoprotein (LDL), alanine aminotransferase (ALT), and blood urea nitrogen (BUN) were determined by enzymatic methods with commercial kits (Biovision Research Products, Inc., CA, USA).

### Histological analysis

Livers from representative mice in each group were fixed in 10% buffered formalin, embedded in paraffin, and cut into 8-μm-thick sections. Some sections were then stained with hematoxylin and eosin (H&E) for histological examination of fat droplets. Images were acquired using an SZX10 microscope (Olympus, Tokyo, Japan).

### Western blot

The liver tissues were homogenized in commercial lysis buffer PRO-PREP® (Intron Biotechnology Inc, Gyeongi-do, Republic of Korea) and then incubated for 25 min on ice to induce cell lysis. The samples were centrifuged at 13,000 rpm (4°C) for 5 min, and the supernatant was transferred to a new 1.5 mL tube. The protein concentration was determined using the Bio-Rad protein assay reagent according to the manufacturer’s instructions (Bio-Rad, Hercules, CA, USA). Protein samples (16 μg) were electroblotted onto a poly-vinyl difluoride (PVDF) membrane following separation on a 10% sodium dodecyl sulfate (SDS)-polyacrylamide gel. Membranes were blocked with a 5% skim milk solution at 4°C for 1 h. The membranes were incubated overnight with anti-SREBP-2 (Santa Cruz, CA, USA) as the primary antibody. Blots were washed 3 times with Tween 20/Tris-buffered saline (TTBS). After the blots were incubated with the corresponding secondary antibody (Santa Cruz, CA, USA) for 1 h at room temperature, they were washed 3 times with TTBS and were then developed using enhanced chemiluminescence (ECL) on X-ray film (Amersham Life Science, Buckinghamshire, UK).

### Real-time polymerase chain reaction (PCR) analysis

Each liver and aorta sample was homogenized, and total RNA was isolated using the Easy-Blue® Reagent (Intron Biotechnology Inc, Gyeongi-do, Republic of Korea) according to the manufacturer’s instructions. Total RNA was quantified using an Epoch® micro-volume spectrophotometer system (BioTek Instruments, Inc. Winooski, VT, USA). In brief, total RNA from the liver and aorta samples was converted to cDNA by using a high-capacity cDNA reverse transcription kit (Applied Biosystems, Foster City, CA, USA). Reverse transcription was conducted in a thermocycler (Gene Amp® PCR system 9700, Applied Biosystems) with the following program: initiation for 10 min at 25°C, followed by incubation at 50°C for 90 min and at 85°C for 5 min. The cDNA synthesized was then stored at −20°C. Real-time PCR analysis was conducted using a Step One Plus® Real-time PCR system (Applied Biosystems). SYBR® Green master mix and primers were used for PCR analysis of glyceraldehyde-3-phosphate dehydrogenase (GAPDH), sterol regulatory element-binding protein 2 (SREBP-2), liver X receptor (LXR), low-density lipoprotein receptor (LDLR), and 3-hydroxy-3methylglutary-CoA (HMG-CoA) in liver tissue and for GAPDH, tumor necrosis factor-α (TNF-α), interleukin-6 (IL-6), intercellular adhesion molecule-1 (ICAM-1), vascular cell adhesion molecule-1(VCAM-1), transforming growth factor (TGF)-β1, and fibronectin in aorta tissue (Santa Cruz, CA, USA). The PCR cycling parameters were as follows: 10 min at 95°C; 40 cycles of 5 s at 95°C and 45 s at 60°C; and a final melting curve of 15 s at 95°C, 1 min at 60°C, and 15 s at 95°C. All primer sequences and annealing temperatures are shown in Table 1. Gene expression was calculated according to the comparative threshold cycle (Ct) method (Applied Biosystems).

**Table 1:** Primer sequences and PCR conditions

**Gene name**	**Tm (°C)**	**Size (bp)**	**Sequence 5′-3′**
Sterol regulatory element binding transcription factor 2	55	177	F: GGCCTCTCCTTTAACCCCTT
			R: CACCATTTACCAGCCACAGG
(SREBP2)			
Liver X receptor	55	119	F: TCCTACACGAGGATCAAGCG
(LXR)			R: AGTCGCAATGCAAAGACCTG
Low density lipoprotein receptor	55	173	F: GCGTATCTGTGGCTGACACC
(LDLR)			R: TGTCCACACCATTCAAACCC
3-hydroxy-3methylglutary-CoA	55	151	F: GTGGCAGAAAGAGGGAAAGG
			R: CGCCTTTGTTTTCTGGTTGA
(HMG-CoA)			
Tumor necrosis factor alpha	55	275	F: AACATCCAACCTTCCCAAACG
(TNFα)			R: GACCCTAAGCCCCCAATTCTC
Interleukin 6	55	162	F: TTGCCTTCTTGGGACTGATG
(IL-6)			R: CCACGATTTCCCAGAGAACA
Intercelluar adhesion molecule 1	55	176	F: GTGGGTCGAAGGTGGTTCTT
(ICAM1)			R: GCAGTTCCAGGGTCTGGTTT
Vascular cell adhesion molecule 1	55	163	F: CTCAGGTGGCTGCACAAGTT
(VCAM1)			R: AGAGCTCAACACAAGCGTGG
Transforming growth factor beta1	55	169	F: GCGGCAGCTGTACATTGACT
(TNFβ)			R: CCGGGTTGTGTTGGTTGTAG
Fibronectin 1	55	197	F: AGCTTTTCCCTGCACCTGAT
			R: GGTGGGTGTCACCTGACTGA

### Statistical analysis

All the values reported have been expressed as the mean ± SEM values for 10 mice. Data were analyzed using one-way analysis of variance (ANOVA) with Dunnett’s test. Statistical analysis was performed using GraphPad Prism (version 5).

## Results

### Effects of SHSST on body weight and food intake

**Figure 1:**
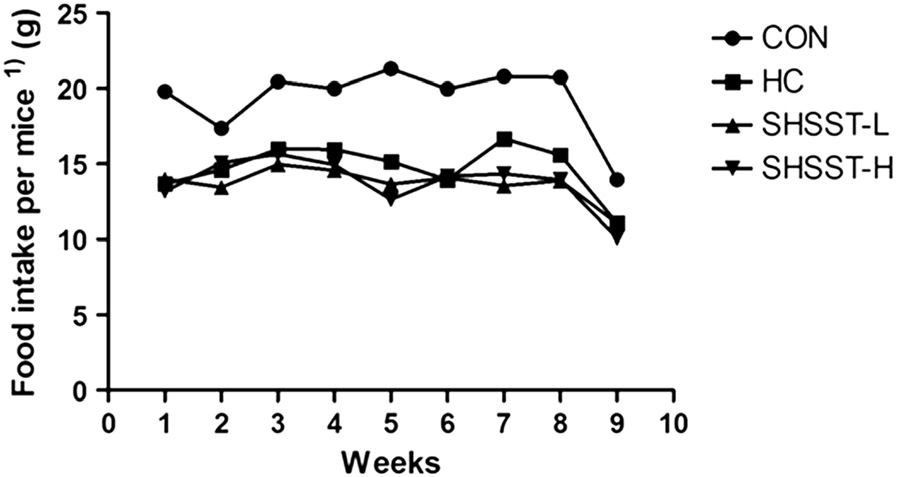
**Effects of SHSST on food intake.** Values have been expressed for 10 mice per group. ^1)^Food intake per mouse = amount of total food intake in each group (g)/number of mice in each group.

Figure 1 shows the food intake of each group. There were no significant differences between the HC group and the SHSST-treated group with regard to food intake. Table 2 shows the total body weight of mice at the starting and final points of the experiments. Although the average body weight of each group was almost the same at the starting point, the body weights in the SHSST-treated group were slightly lower than those in the HC group in the final week.

**Table 2:** Body weights (g) at starting week and final week respectively

	**CON**	**HC**	**SHSST-L**	**SHSST-H**
Start week (0)	22.49 ± 1.08	22.13 ± 0.95	22.72 ± 1.37	22.9 ± 1.09
Final week (9)	25.75 ± 1.12	25.7 ± 1.12	23.76 ± 2.22	23.7 ± 0.92

Values have been expressed as the mean ± SEM values of 10 mice per group.

### Effects of SHSST on TC and LDL

**Figure 2:**
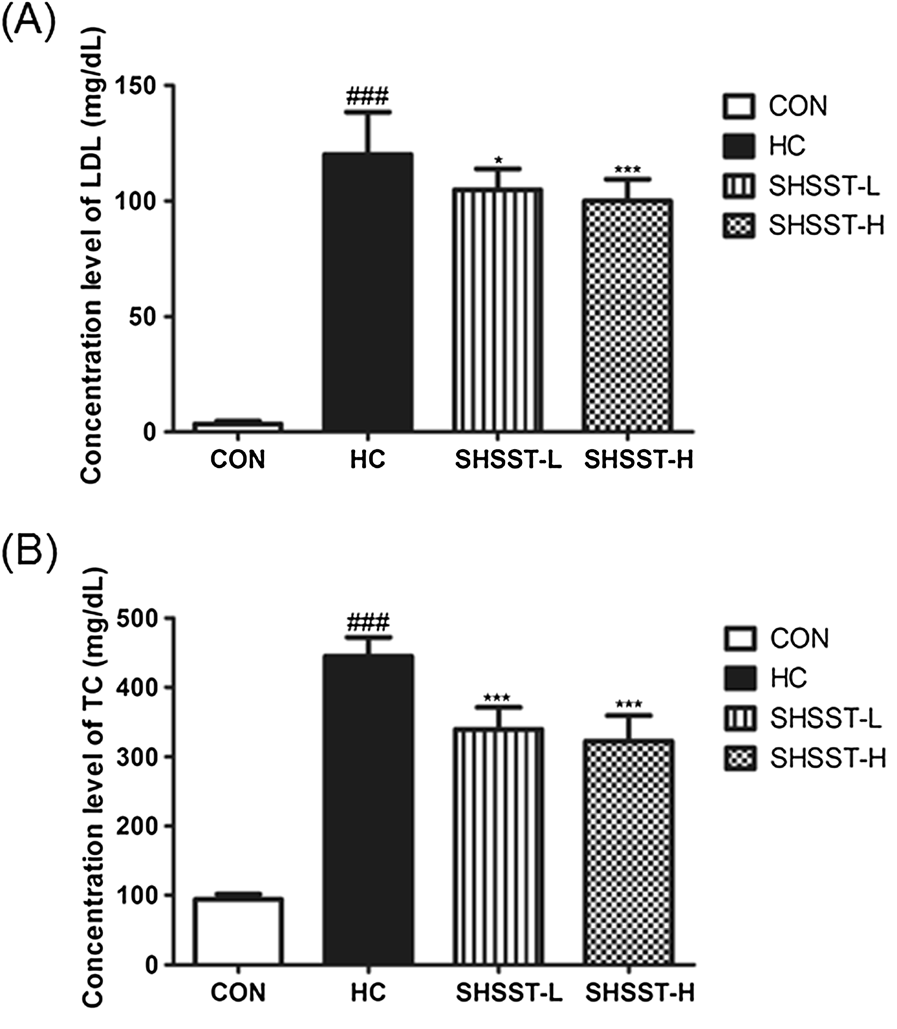
**Effects of SHSST on LDL and TC in experimental mice. (A)** Serum level of LDL **(B)** Serum level of TC, CON, standard diet group; HC, high cholesterol diet group; SHSST-L, HC plus 30% ethanol extract of SHSST treatment group, SHSST-H, HC plus 80% ethanol extract of SHSST treatment group. Values have been expressed as the mean ± SEM values of 10 mice per group. ^###^*p* < 0.001 vs. CON group. ^*^p < 0.05 vs. HC group, ^***^p < 0.001 vs. HC group.

Figure 2 shows the concentrations of serum TC and LDL in each group. The concentrations of TC and LDL were higher in the HC group than in the other groups. Administration of SHST significantly suppressed elevation of the serum TC and LDL levels. In the SHSST-H group, although there was no statistical significance between the results for SHSST-L and SHSST-H, the TC and LDL levels were lower than those in the SHST-L group.

### Effects of SHSST on ALT and BUN

**Figure 3:**
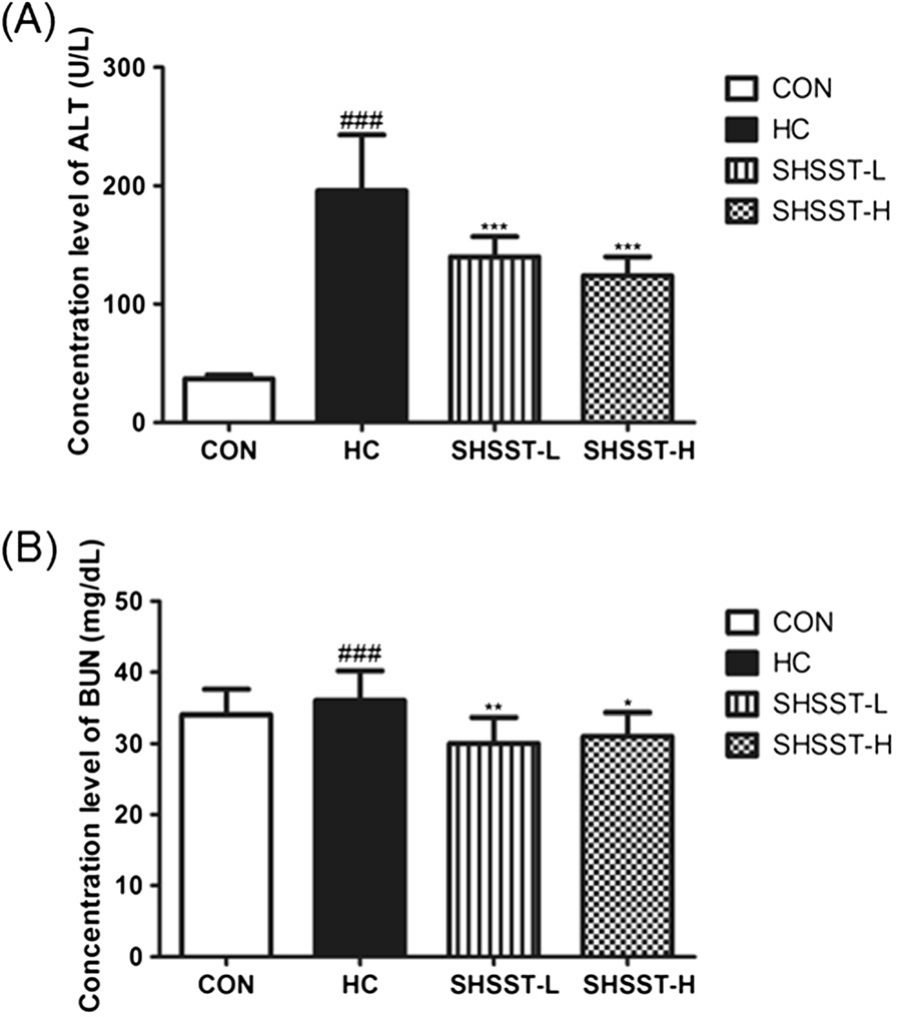
**Effects of SHSST on ALT and BUN in experimental mice. (A)** Serum level of ALT **(B)** Serum level of BUN. CON, standard diet group; HC, high-cholesterol diet group; SHSST-L, HC plus 30% ethanol extract of SHSST treatment group; SHSST-H, HC plus 80% ethanol extract of SHSST treatment group. Values have been expressed as the mean ± SEM values of 10 mice per group. ^###^*p* < 0.001 vs. CON group. ^*^p < 0.05 vs. HC group, . ^**^p < 0.01 vs. HC group, ^***^p < 0.001 vs. HC group.

The concentrations of ALT and BUN were higher in the HC group than in any of the other groups (Figure 3). In addition, the level of ALT in the SHSST-H group was lower than that in the SHSST-L group. In contrast, the level of BUN in the SHSST-L group was lower than that in the SHSST-H group. In particular, the level of BUN in the SHST-treated groups was slightly lower than that in the CON groups, although no statistically significant difference was observed between the SHSST-L group and SHSST-H group. In addition, all toxicity markers in the SHSST-treated group were within the normal range.

### Histological examinations

Figure 3 shows the H&E staining results for liver tissues from each group. In the HC group, lipid droplets appeared as small vacuoles within the liver cells near veins. Enlargement of lipid droplets in the liver tissue of mice in the HC group was more pronounced than that in the SHST-treated groups. Thus, the results for each representative tissue clearly showed that lipid accumulation in the liver was more considerable in the HC group than in the SHSST-L and SHSST-H groups.

### Effects of SHSST on liver tissue

**Figure 4:**
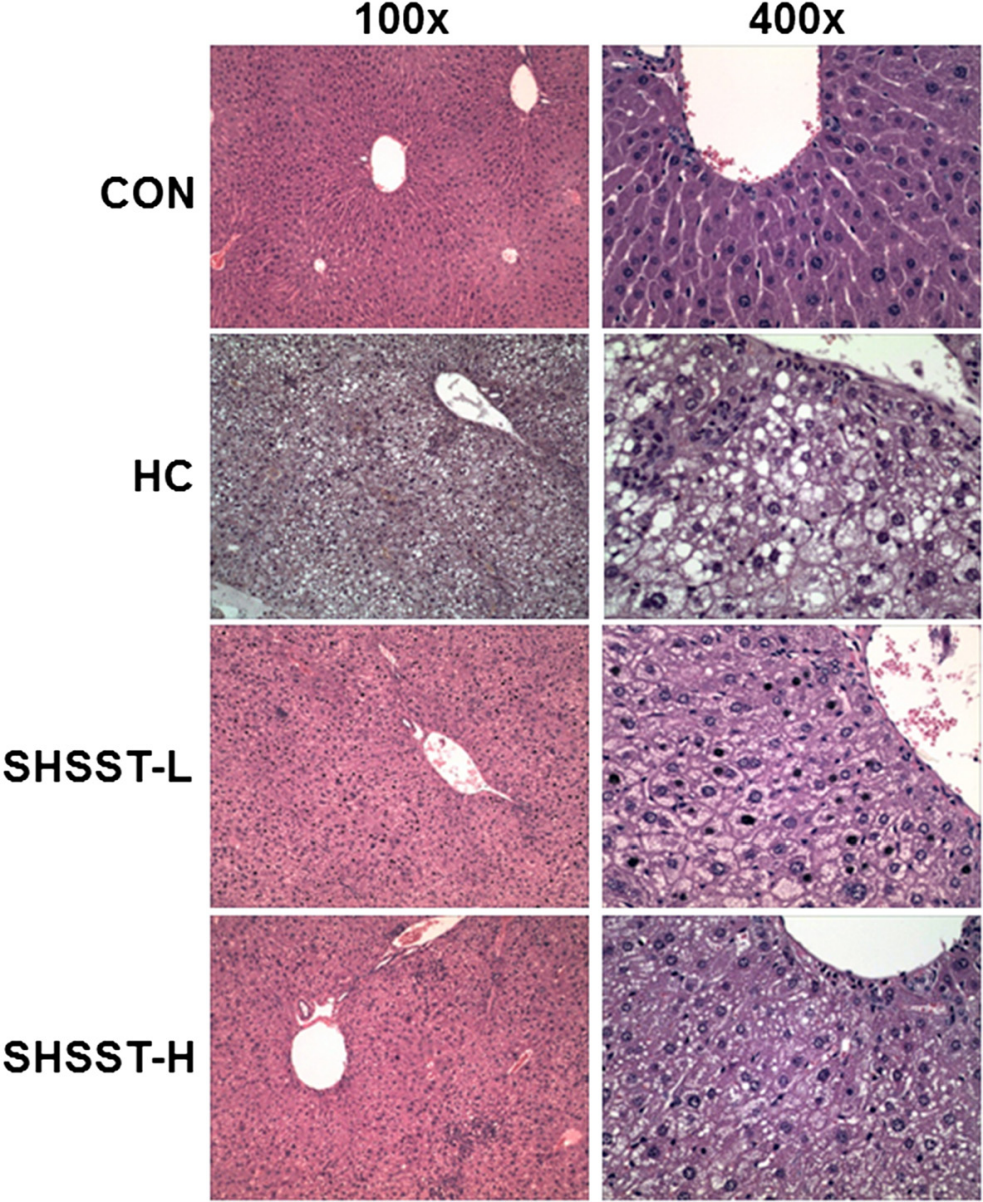
**Effects of SHSST on representative H&E staining of liver tissue.** Pictures in the left panel are at the original magnification of 100×. The pictures in the right panel are at the original magnification of 400 × .

**Figure 5:**
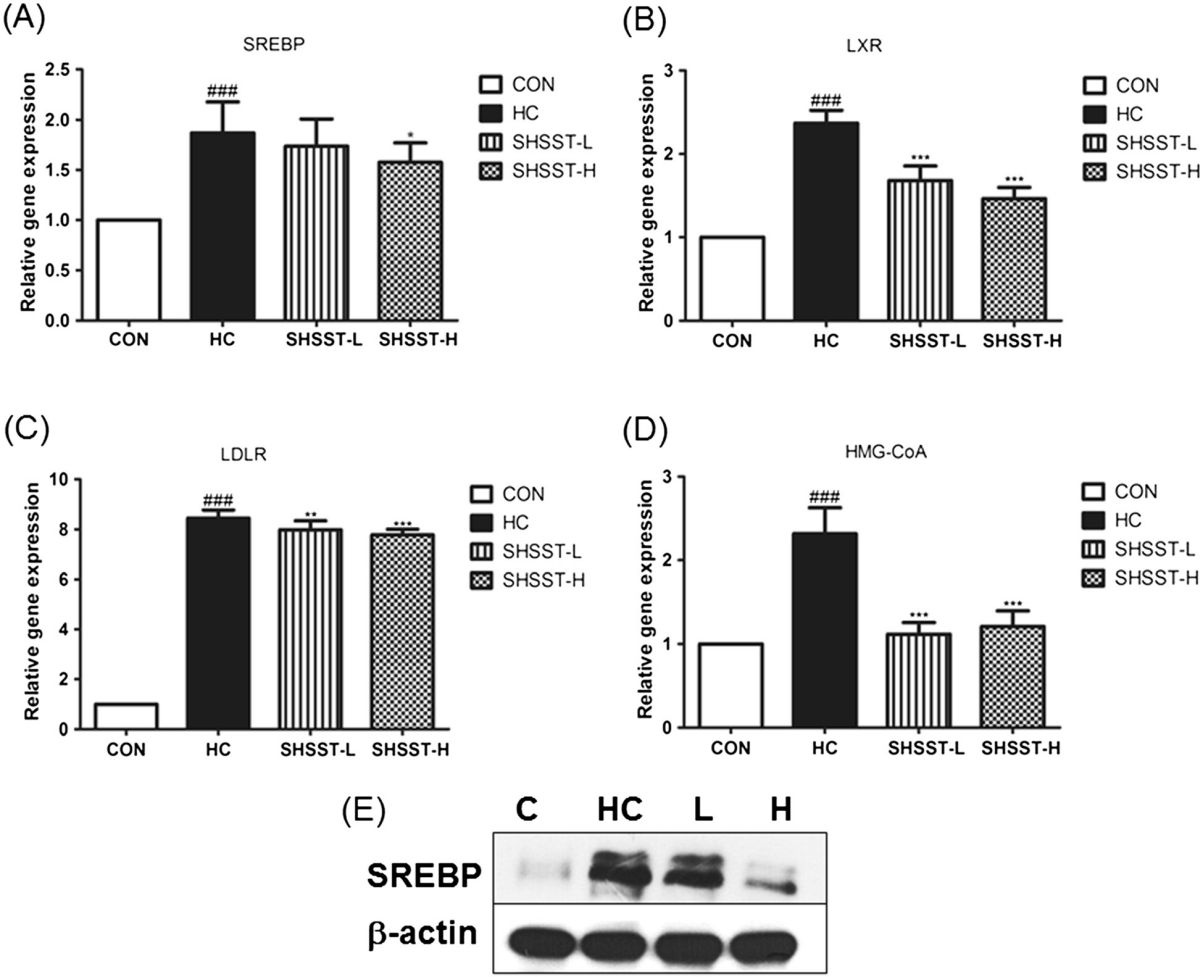
**Effects of SHSST on liver tissue. (A)** mRNA expression of SREBP-2, **(B)** LXR, **(C)** LDLR, and **(D)** HMG-CoA. **(E)** was Western bolt of SREBP-2. Data were normalized to GAPDH mRNA expression levels and then compared to the values for the CON group, which were assigned a value of 1.0. CON, standard diet group; HC, high-cholesterol diet group; SHSST-L, HC plus 30% ethanol extract of SHSST treatment group; SHSST-H, HC plus 80% ethanol extract of SHSST treatment group. Each bar represents the mean ± SEM value of 2 independent experiments for 10 mice from each group. ^###^*p* < 0.001 vs. control group, ^*^p < 0.05 vs. HC group, ^**^p < 0.01 vs. HC group, ^***^p < 0.001 vs. HC group.

Figure 4 shows the results of western blot analysis. Protein expression of SREBP2, which is a key transcription factor involved in cholesterol homeostasis, was downregulated in the SHSST-treated group relative to that in the HC group. To determine whether the reduced amount of lipid droplets in SHSST-treated mice was associated with expression of hepatic genes involved in lipid and cholesterol metabolism, we examined the expression of SREBP2, LXR, LDLR, and HMG-CoA. In liver tissue, the mRNA expression of SREBP, LXR, LDLR, and HMG-CoA was clearly lower in the STSST-treated groups than in the HC group (Figure 5). In addition, the expression of SREBP2, LXR, and LDLR in the SHSST-H group was lower than that in the SHSST-L group.

### Effects of SHSST on aorta tissue

**Figure 6:**
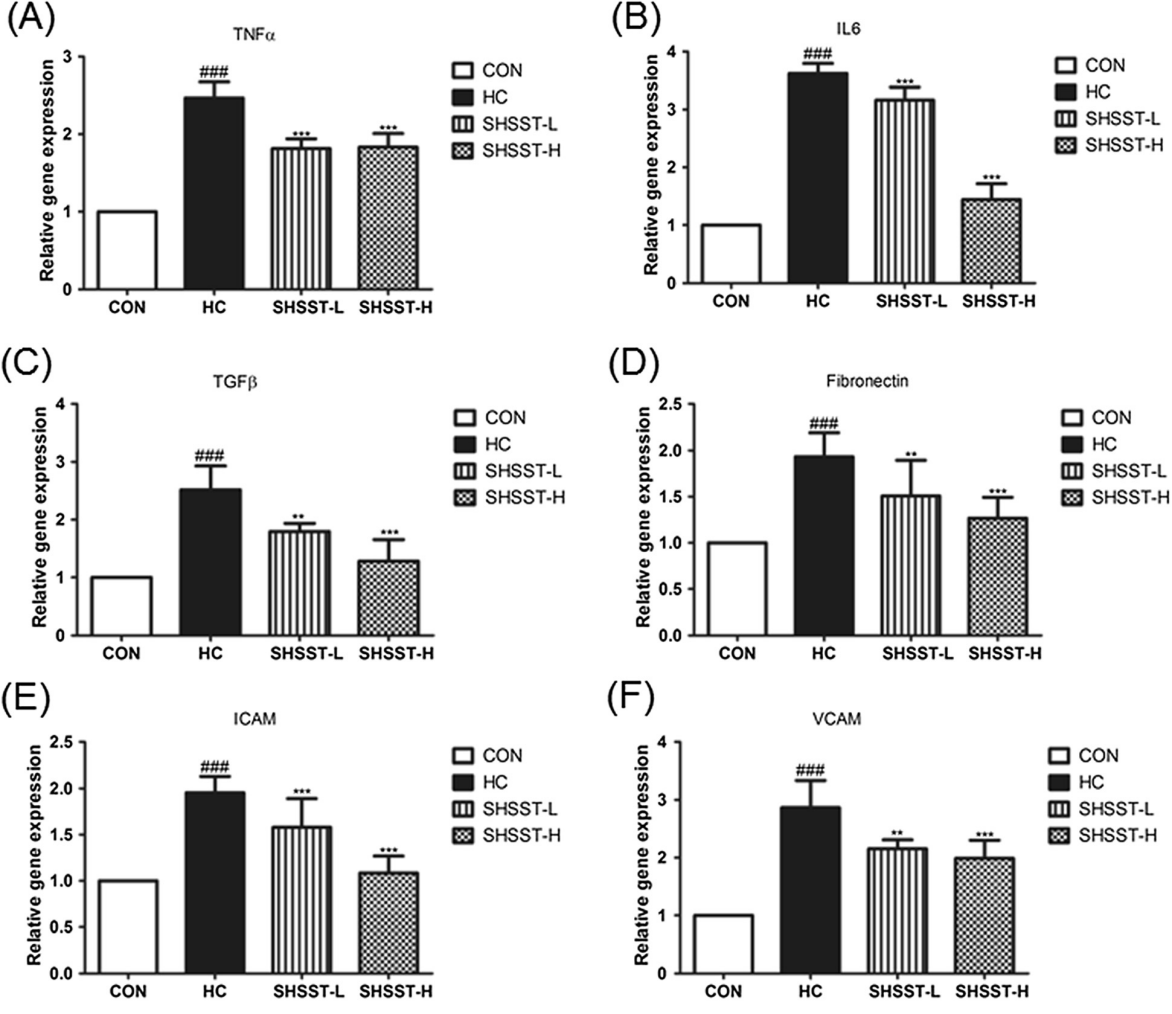
**Effects of SHSST on aorta tissue.** mRNA expression of **(A)** TNF-a, **(B)** IL-6, **(C)** TGF-b, **(D)** Fibronectin, **(E)** ICAM, **(F)** VCAM. Data were normalized to the GAPDH mRNA levels and then compared to the values for the CON group, which were assigned a value of 1.0. CON, standard diet group; HC, high-cholesterol diet group; SHSST-L, HC plus 30% ehtanol extract of SHSST treatment group; SHSST-H, HC plus 80% ethanol extract of SHSST treatment group. Each bar represents the mean ± SEM values of 3 independent experiments for 10 mice from each group. ^###^*p* < 0.001 vs. control group, ^**^p < 0.01 vs. HC group, ^***^p < 0.001 vs. HC group.

The mRNA expression of several molecules in aorta tissue is shown in Figure 6. In the HC group, the mRNA expression of all the molecules was higher than that in any other group, and SHSST administration significantly suppressed this elevation in mRNA expression. In addition, the mRNA expression of these molecules in the SHSST-H group tended to be lower than that in the SHSST-L group.

## Discussion

NAFLD represents a histological spectrum of liver disease related to diabetes, insulin resistance, and obesity; this spectrum largely extends from isolated hepatic steatosis to steatohepatitis and cirrhosis. Hepatic steatosis is not only one feature of NAFLD but also both a potential cause of advanced liver disease and is also considered to be an important cofactor in the pathogenesis of various other liver diseases [21,22]. The development of hepatic steatosis is known to be closely associated with genetic and environmental factors [7,23,24].

The liver plays a central role in cholesterol and lipid metabolism [25]. It is widely assumed that cholesterol metabolism is governed by several hepatic genes, including SREBP-2, LXR, LDLR, and HMG-CoA [26,27]. In particular, members of the SREBP family such as SREBP1a, SREBP1c, and SREBP2 are key transcription factors that are known to control the production of lipids for export into the bile as micelles and into the serum as lipoproteins [28]. Therefore, the protective effects of SHSST against liver steatosis may be studied by measuring suppressing of the expression of those several key hepatic transcription, especially SREBP-2.

Chronic intake of a high-fat diet has been reported to cause low-grade inflammation. In addition, excessive intake of cholesterol may cause vascular inflammation, and pro-inflammatory cytokines such as TNF-α and IL-6 may stimulate the expression of adhesion molecules and chemokines such as VCAM-1, ICAM-1, and fibronectin in aorta tissue [29-31]. VCAM-1 and ICAM-1 are thought to play an important role in the process of atherosclerosis by recruiting inflammatory cells, and they are both are upregulated by pro-atherogenic factors [31].

In the present study, we investigated the protective effect of SHSST against hepatic steatosis in mice with HCD-induced hepatic steatosis. We also used 2 different types of SHSST extracted using 30% and 80% ethanol to determine effects of extraction conditions on the efficacy of SHSST. The reason we decide to study effect of SHSST on hepatic steatosis specifically is simple and clear: as we had mentioned before, one of major factor causing NAFLD is over-substitution of high fat or high cholesterol. Though, NAFLD is a very comprehensive subject, hepatic steatosis is one of simple and basic features of NAFLD. To researchers, it means that demonstrating effect of SHSST on hepatic steatosis is more efficient way to demonstrate positive effect of SHSST related with lipid metabolism in liver specifically. That is why we decided to study SHSST in liver steatosis induced by HCD. SHSST has various active compounds, such as baicalein, baicalin, wogonin, and berberine. Baicalein has been reported that it can ameliorate obesity, dyslipidemia, fatty liver, and diabetes and insulin resistance [32]. Bak et al., reported Wogonin have beneficial effects on glucose and lipid metabolism related to enhanced PPARα and adiponectin expression via AMPK activation [33]. Berberine has effects on NAFLD, diabetes, and hyperlipidemia [34]. We can suggest that these components from 3 herbs may be candidates against hepatic steatosis. The main compound for inhibiting hepatic steatosis has to investigate more.

In our study, SHSST clearly suppressed serum TC and LDL levels relative to those in the HC group. The protein levels of SREBP, a key transcription factor in cholesterol metabolism, were also lower in the SHSST-treated group than in the HC group. In addition, SHSST significantly inhibited the hepatic mRNA expression of several molecules such as SREBP-2, LXR, LDLR, and HMG-CoA that govern cholesterol metabolism, and it also suppressed the mRNA expression of TNF-α, IL-6, VCAM-1, ICAM-1, and fibronectin in aorta tissue, relative to that in the HC group. These findings suggest that SHSST protected the liver from hepatic steatosis and aorta tissue from the initiation of atherosclerosis.

In our study, the SREBP-2 expression in protein level and mRNA expression of several molecules in the liver and aorta was inhibited to a greater extent in the SHSST-H group than in the SHSST-L group, which suggests that the effective component of SHSST may be more efficiently extracted by using a high percentage of ethanol. It is interesting results. However, this is more than nothing but local observations and precise studies are needed to investigate the different pharmacological components between SHSST-L and SHSST-H. We expect that this issue might be solved in the near future.

The serum levels of ALT and BUN, which are specific toxicological markers, are enhanced in the case of liver injuries and kidney injuries, respectively [35,36]. In the present study, SHSST significantly decreased the increase in ALT and BUN levels, relative to those for the HC group. Thus, SHSST protected the liver and kidney from possible damage that could resulted from excessive intake of dietary cholesterol.

## Conclusions

Our current investigation clearly demonstrates the protective effects of SHSST against hepatic steatosis by inhibiting the mRNA expression of key hepatic molecules such as SREBP-2, LXR, LDLR, and HMG-CoA. Our results also suggest that SHSST may suppress the initiation of atherosclerosis. These findings shed light on a possible preventative measure against liver steatosis.

## Competing interests

The authors declare that they have no competing interests.

## Authors’ contributions

Conceived and designed the experiments: YBK. Performed the experiments: TGA, SYC, and JYL. Analyzed the data: HJA. Contributed reagents/materials/analysis tools: HJA, and YBK. Wrote the paper: TGA and JYL. All authors read and approved the final manuscript.

## Pre-publication history

The pre-publication history for this paper can be accessed here:

http://www.biomedcentral.com/1472-6882/13/366/prepub
